# An Object Lesson in Hygiene

**Published:** 1894-04

**Authors:** 


BUFFALO MEDICAL AND SURGICAL JOURNAL
A MONTHLY REVIEW OF MEDICINE AND SURGERY.

*EDITORS:*

THOMAS LOTHROP, M. D. - - WM. WARREN POTTER, M. D.

All communications, whether of a literary or business character, should be addressed to the managing editor: 284 FRANKLIN STREET, BUFFALO, N. Y

VOL. XXXIII. APRIL, 1894. NO. 9.

### AN OBJECT LESSON IN HYGIENE.

OUR servants and protectors in two of the more important departments of our municipal government have given the medical profession of the city a rare opportunity to study a sanitory problem of singular interest and importance, and upon a scale of singular magnitude. In order to fully realize the significance of the sanitory situation of our city during the first week of March, we must glance for a moment at the habitual experience of the people of our State during the last ten years with the malady, typhoid fever. In brief, this disease prevails throughout the State and during the entire year, having each year a distinct period of increase from May to November, and a definite and regular period of decline from November to May; the months August, September and October habitually furnishing four times as many deaths as the months March, April and May.

The testimony of our colleagues, whether in private or hospital service, seems to set forth most conclusively that our experience of this disease was the usual observation during the months of December, January and February, and the same testimony as clearly establishes that our experience with typhoid fever during the month of March, 1894, is alarming, is a complete reversal of the legitimate expectation of the month, and is utterly unprecedented in the history of this city.

Typhoid fever is epidemic in Buffalo, and has been since March 1st. Up to this writing over 450 cases have been reported, and of these undoubtedly over 200 fell ill in one week,—the first week of March. From inquiry we learn that the disease knows no locality within our city limits, save one to be named later, and is absolutely independent of hygienic or unhygienic environment, claiming its victims at will from the hovel of the tramp, the cottage of the laborer and the palace of the politician.

Over against this unseasonable and furious outbreak of infectious disease stands the following fact of terrible significance: At various times during the month of February of this year the supply of water in the city reservoir was not more than one-half or one-quarter of the usual quantity. In order to increase this deficient supply, water was pumped from a shallow portion of the river near its margin, where its slow moving currents are habitually befouled by the sluggish and sewage-laden waters of the Buffalo harbor, the two being at this place only nominally divided, and that by a slender wall of loose masonry,—the Bird island pier. Our only real protectors, the city press, seem unable to fix accurately the dates on which the poison-laden, death-dealing waters of the harbor were substituted for the pure Niagara, of which we are so justly proud; various dates have been mentioned, and, so far as we know, not contradicted by those whose business it is to know, and who may yet be made to tell. February 1st, 5th, 12th, 16th, 21st and each of the remaining days of the month are thus mentioned.

The precise facts regarding the occasions when our water supply was polluted, or the exact proportions of poisoned and pure water which our servants of the Water Department furnished for our drinking, may never be known, but no physician at all familiar with literature as to the causation of typhoid fever, can for a moment doubt the causative relation of our befouled water of February with the outburst of disease of the first days of March. Cause was never more plain, and effect never more unmistakable. And yet we have not heard of any shootings, hangings or burnings. Were we quite sure that this apparent composure, forbearance and self-control, exhibited by the masters of the house (whose well-paid servants have just shown their utter want of either capability, efficiency or loyalty by a wholesale poisoning), was not in good part merely the result of ignorance and scepticism, we should say that Christian forgiveness and charity has never shown more benignly than in the disease-stricken families of Buffalo this March, 1894.

We remarked that the present epidemic knew no locality within our city limits save one,—namely, the area supplied by water from the Jubilee Water Works, which water comes from pure springs. From this territory of about 260 dwellings comes but a single case of the disease.

Our people maintain at great expense a water bureau and a health department, to the end that we may enjoy the advantage of a free supply of pure water,—the article of food second in importance only to pure air,—and to the further end that all possible care, diligence and knowledge may be exercised with ceaseless activity for the protection of the public health. In the name of the medical profession and in the interest of the public, we would like to ask our servants in these two offices certain questions:

1. How far apart in space or time are the headquarters of the Board of Public Works and the Health Departments?

2. How frequently and when during the months of December, January and February were consultations held between these departments upon the question of abandoning the reservoir on Niagara street, of opening the reservoir on Best street and of opening the inlet at Bird island pier?

3. What records are on file in the respective offices of any such consultations and the resulting advices and recommendations?

4. What care and pains were taken to clean the reservoir on Best street before filling the same with water?

5. How far above low water mark,—the bottom of the river,—are the inlets to the new tunnel?

6. How far above low water mark, as above indicated, are the bottoms of the suction pipes in the wells at the pumping station?

7. On what days in February, 1894, was water pumped from the inlet of Bird island pier?

8. While water was being pumped from the Bird island pier inlet, was Niagara water also being pumped, and what were the relative quantities of each?

9. Does the Bird island pier inlet lead to the pumping station through the old or the new tunnel?

10. In case the Bird island inlet leads to the pumps through the old tunnel, what means were used to clean the same before pumping from it for city use?

11. When and how did the Health Department first learn that water was being furnished the city for domestic use from Bird island inlet?

12. What steps, if any, did the Health Department take to ascertain the reason why foul water was furnished the city at various times during the month of February, 1894?

13. To whom do the incumbents of these departments owe allegiance,—to the politicians who gave them place or to the people who pay the taxes ?

